# Goodwill impairment, M&A, and industry development—Empirical evidence from listed companies in China

**DOI:** 10.1371/journal.pone.0290442

**Published:** 2023-08-24

**Authors:** Yadi Wen

**Affiliations:** School of Accounting, Southwest University of Finance and Economics, Chengdu, Sichuan Province, China; University of City Island, CYPRUS

## Abstract

Goodwill has been a controversial issue in China since 2007 when the new accounting standards changed the subsequent measurement of goodwill from periodic amortization to impairment testing. Using the change in subsequent measurement of goodwill, this paper examines the impact of goodwill accounting on corporate M&A and industry development. The paper finds that adopting the goodwill impairment test significantly increases firms’ M&A incentives, as evidenced by a significant increase in the frequency and probability of M&A and a significant reduction in the time interval between successive M&A at the industry level. From an industrial perspective, the impairment policy has significantly improved industry concentration, total factor productivity, and competitive advantage in international trade across Chinese industries. The positive impact of goodwill impairment policy on M&A is more pronounced among firms with asset-light characteristics or high P/E ratios, and its contribution to industry competitiveness is more pronounced in asset-light or high P/E ratios industries. From the perspective of promoting capital market mergers and acquisitions and industry competitiveness, the article finds that the current goodwill impairment policy can have positive economic consequences. Our study breaks through existing perspectives to provide compelling empirical evidence for the current theoretical and practical controversy over goodwill measurement standards.

## Introduction

In recent years, the phenomenon of listed companies taking hefty goodwill impairment charges leading to negative earnings has occurred frequently, especially in 2018, when the goodwill impairment charges of China’s A-share listed companies reached a new record high of 162.5 billion yuan in total, of which 255 loss-making companies took goodwill impairment charges of 141.5 billion yuan. Goodwill risk seems to have become a "sword of Damocles" in China’s capital market, causing concern and reflection on the accounting treatment of all sectors of society. In November 2018, the China Securities Regulatory Commission issued the document "Accounting Regulatory Risk Alert No. 8—Goodwill Impairment", pointing out that the exposure to goodwill impairment risk may have a significant impact on the company’s actual operating results, and drawing attention to the accounting regulatory risks related to the subsequent measurement of goodwill, making the subsequent measurement of goodwill a core issue of goodwill accounting policy.

Since 2007, Chinese accounting standards have required goodwill to be recognized as the excess of the purchase price paid over the fair value of the net assets of the acquired business on M&A and impairment tests to be performed at the end of each year to recognize losses. The goodwill balances (The goodwill impairment charges of China’s A-share listed companies) have increased rapidly since 2007, but the average goodwill impairment rate (The ratio of the total goodwill impairment charges to the goodwill balances of listed companies in the year) before 2017 was less than 2%, indicating that Chinese listed companies are generally less willing to impair goodwill. However, in 2018, listed companies took huge goodwill impairment charges, with an impairment ratio of 12.5%, and the ratio of goodwill impairment to net profit for 2018–2019 was even over 50%, and the vast goodwill impairment seriously eroded the earnings. This trend suggests that listed companies may be using goodwill impairment to take a "big bath", and many studies have argued that the current goodwill impairment standard is a tool for companies to manage earnings or manipulate financial data [1]. In the context of the goodwill bubble and impairment anomalies, in January 2019, the Accounting Standards Board of the Ministry of Finance issued an "Accounting Standards Update", in which the advisory members considered it more reasonable to write down goodwill amortization to zero as compared to goodwill impairment.

However, it is limited to assessing the impact of goodwill impairment policies only at the quality of traditional accounting information level, as the ultimate objective of accounting standards is to optimize the efficiency of resource allocation and improve economic performance. First, eliminating goodwill impairment does not directly constrain management’s opportunistic behavior, and firms with excess managerial motives will still achieve their goals by other means [2]. Secondly, the change to direct amortization does not reflect the nature of the goodwill asset and does not reduce the risk of the current high level of goodwill. Accounting standards not only provide high-quality accounting information and thus influence the investment efficiency of capital market investors but also directly impact the investment activities and operational efficiency of enterprises. In a technological age, the importance of intangible assets, represented by human capital, technology, and brands, is growing. But most of these are not recognized as assets in accounting because they cannot be valued accurately and effectively, resulting in vast amounts of goodwill that inevitably arise from M&A activity. At the same time, M&A is a fundamental factor in allocating market resources and promoting national economic development and international competitiveness [3]. Therefore, the subsequent measurement of goodwill is an important economic issue affecting the success of corporate M&A and industrial development. The formulation of this accounting standard needs to be considered from the perspective of the resource allocation efficiency of the entire capital market.

Some studies support the positive effects of goodwill impairment: goodwill impairment can convey effective messages to investors and can meet companies’ needs for sustainability through continued M&A [4–6]. However, some studies have also shown that most M&As often fail to improve company performance and create value, suggesting that the goodwill impairment that encourages M&A activities can lead to worse economic consequences [7]. At this point, goodwill impairments resulting in high premium M&As can harbor hidden risks for the capital market. From international experience, the six waves of global M&A are the main ways for developed countries to accelerate industrial layout, promote the industrialization process and enhance national competitiveness. The US capital market, led by the new economy, has seen rapid development in its M&A activity and rising goodwill balances after the change in goodwill accounting from amortization to impairment, with goodwill balances reaching $4.2 trillion and goodwill impairments reaching $71 billion at the end of 2019. The high level of goodwill in the US is mainly due to technology assets in new economy industries, which have enabled the US to maintain a leading position in fierce international competition. Therefore, this paper attempts to comprehensively examine the economic consequences of the goodwill impairment standard from the perspective of Chinese firms’ M&A behavior and industrial competitiveness.

The new accounting standards implemented in China in 2007 changed the previous practice of goodwill amortization. They introduced impairment testing for the subsequent measurement of goodwill, providing a research opportunity with quasi-natural experimental characteristics to empirically demonstrate the impact of goodwill impairment policy on corporate M&A activities and industrial competitiveness. This paper finds that: (1) The adoption of impairment tests increases firms’ M&A enthusiasm, as evidenced by significantly higher M&A probabilities, higher M&A frequencies, and shorter consecutive M&A intervals; (2) The goodwill impairment policy further enhances industrial competitiveness by improving industry concentration, production efficiency and comparative advantage in international industrial trade; (3) The positive impact of goodwill impairment on M&A is more pronounced for asset-light or high P/E companies with more robust M&A demand and higher M&A premiums, while goodwill impairment also improves industrial competitiveness more for asset-light or high P/E industries. These findings hold after robustness tests such as time trends and replacement variables.

The contributions of this paper: First, existing research mainly negates the goodwill impairment policy from the perspective of accounting information quality. This paper effectively examines the economic consequences of the subsequent measurement of goodwill from the perspective of promoting both corporate M&A and industrial competitiveness, supporting the positive impact of goodwill impairment accounting policies on resource allocation and economic development. It argues that the ongoing measurement of goodwill through impairment testing should be continued. Second, the results of this paper suggest that accounting policies significantly influence corporate investment and industrial development, expanding the field of research on the factors influencing M&A behavior. Third, the findings of this paper provide valid empirical evidence on the current theoretical and practical controversies over goodwill measurement standards. It is an essential reference for relevant institutions in developing and regulating accounting standards for the measurement of goodwill as well as other assets.

## Literature review and research hypothesis

### Institutional background review and literature review

Table 1 shows that China, the US, and the International Accounting Standards Board (IASB) have all gone through a process of changing their policies on the subsequent measurement of goodwill from amortization to impairment.

**Table 1:** Treatment of China, the US, and IASB policies on subsequent measurement of goodwill.

	The period of amortization	The period of impairment testing
**China**	1993–2006	2007-present
	PRC ASBE: Goodwill is an intangible asset amortized over 10 years.	PRC ASBE: Goodwill is a separate asset and should be subject to an impairment test annually.
**United States**	1970–2000	2001-present
	GAAP: The accounting for business combinations is divided between the pooling-of-interests method and the purchase method, with goodwill arising under the purchase method being amortized over 40 years.	GAAP: Abolition of the pooling-of-interests method and requirement to test goodwill for impairment
**IASB**	1983–2003	2004-present
	IAS: The accounting for business combinations is divided between the pooling-of-interests method and the purchase method, with goodwill arising under the purchase method being amortized over 20 years.	IFRS: Abolition of the pooling-of-interests method and requirement to test goodwill for impairment

Goodwill was standardized for accounting purposes in the US as early as 1970. With the development of M&A activity, the value of goodwill as an asset was supported by literature that goodwill can effectively enhance the value of capital and is one of the core competencies of modern companies [8, 9]. Then, in the 1990s, with the rapid development of the US technology industry, the share of goodwill in the total assets of listed companies began to rise, and the disadvantages of goodwill amortization were revealed. The amortization of goodwill has been widely criticized for reducing the quality of accounting and discouraging investment. First, the arbitrariness of the amortization period reduces the quality of accounting information, as companies may manipulate the amortization period based on capital and performance needs, given the significant impact of amortization on profit [10]; The amortization of goodwill is weakly correlated with changes in the value of the company, does not reflect the economic substance of the goodwill and does not provide any new information to the market, which ultimately reduces the efficiency of investors [9, 11]. Second, in the context of the globalization of capital, the goodwill amortization policy puts the US at a disadvantage in international mergers and acquisitions compared to the UK, where goodwill can be charged against equity, and hinders its globalization [12].

With the fifth wave of mergers and acquisitions in the United States, the total amount of goodwill continued to increase. As a result of lobbying by companies, in 2001, the new US accounting standards began to include an impairment test for the subsequent measurement of goodwill. This approach is supported by empirical evidence that goodwill impairment reflects a firm’s economic condition timelier and conveys accurate information about the firm than amortization [13, 14]. In addition, goodwill impairment reduces the impact on firms’ profits, allowing companies to pursue more investment opportunities [15]. Following the introduction of goodwill impairment testing in the United States and the rapid development of new economy industries, the IASB issued a new IFRS in 2004 to ensure the comparability of accounting information in international M&A activities in the context of globalization, which changed goodwill amortization to impairment testing.

The new corporate accounting standards implemented by the Chinese Ministry of Finance in 2007 changed goodwill amortization to impairment testing, thus converging with international accounting standards. However, the existing literature on goodwill accounting in China has mainly rejected this policy from the perspective that it reduces the reliability of accounting information, arguing that goodwill does not reflect the future value of the company when management has discretionary power [16], but instead exacerbates information asymmetries with analysts, auditors and external investors [17–19]. As a result, the majority view in the literature supports abolishing the goodwill impairment policy and returning to the amortization policy. This paper compares firms’ M&A behavior and industry competitiveness before and after the change in goodwill accounting from amortization to impairment and attempts to provide empirical evidence for the positive effects of goodwill impairment testing from the perspective of resource allocation and economic development.

M&A is an essential means of allocating market resources and promoting national economic development, and with the rise of the Chinese economy and the success of social transformation, M&A activities in the Chinese context have attracted substantial attention [20, 21]. The measurement of goodwill is a crucial aspect of M&A accounting activities and can directly impact the performance and success of M&A transactions. Apart from Dunne et al (1998) [22]., who found that different goodwill accounting policies affect post-merger cash flows through case studies in the US, Japan, and the UK, no empirical study examines the impact of goodwill accounting policies on M&A activity in the Chinese scenario. This paper examines the relationship between accounting policies and M&A behavior in an attempt to explore the role of accounting standards in China’s economic development and to broaden the field of research on the factors influencing M&A behavior.

### Research hypothesis

The change in the subsequent measurement of goodwill from amortization to impairment will directly impact the company’s post-acquisition profits. Suppose the company is exempt from goodwill amortization. In that case, post-merger performance will not fluctuate significantly, which can increase the motivation for M&A in the following aspects: First, according to the theory of synergy effect, the company’s motivation for M&A is to achieve operating or financial synergy. If the post-merger performance is not affected by the amortization and the performance is stable, it will not affect stakeholders’ assessment of the acquisition’s performance. It will facilitate the rapid integration of the company to achieve synergies. Adopting the impairment policy will give the company a better expectation of achieving the M&A target, which will enhance the company’s M&A motivation. Second, at the managerial level, Mueller (1969) [23] argues that management has a strong incentive to expand the size of the firm and will try to increase the firm’s profits through M&A to enhance power, prestige, and compensation [24, 25]. Goodwill amortization will cause post-merger performance to fluctuate significantly, inevitably affecting management prestige and remuneration, which in turn will reduce management’s motivation to pursue M&A. Finally, the functional fixation in China’s capital market means that equity or debt investors pay more attention to the impact of significant investment decisions on the accounting earnings than the actual value of the company’s investment [26]. Goodwill amortization reduces the company’s post-M&A accounting profit. In this case, investors will perceive the M&A negatively, which will reduce the company’s market valuation and financing ability and discourage companies from engaging in M&A activities. Therefore, the change in the subsequent measurement of goodwill from amortization to impairment will eliminate the negative impact of M&A goodwill on post-M&A accounting results and will alleviate the concerns of the company itself, its management and external investors about its performance, which in turn will enhance the motivation of the company’s M&A investment activities.

In summary, we propose Hypothesis 1:

H1: The change in the subsequent measurement of goodwill from amortization to impairment will promote the company’s incentive to make M&A.

Impairment of goodwill can reduce the performance pressure of stakeholders in M&A activities, which can promote M&A enthusiasm and help companies achieve their M&A objectives, such as finding resources and improving operational efficiency, and thus have a positive economic impact on industrial development. However, relevant empirical studies have shown that the M&A in China is characterized by hype, fraud, and failure and does not create value for firms [7]. The accounting treatment without mandatory goodwill amortization will undoubtedly catalyze such blind M&A, overpayment, and other M&A problems, harming enterprises’ development and causing worse economic consequences [27].

In contrast, most studies conclude that corporate M&A failures are based on the individual micro level of the company and focus on financial performance. In the new trend of industrial development, as Chinese enterprises face increasingly fierce international competitive pressure, M&A is not only a choice of business expansion but also a strategic tool for industrial competition and economic development. Moreover, from the perspective of resource allocation efficiency, active M&A remains an effective means of industrial upgrading and transformation and is a significant driver of China’s industrial economic development [28]. On the one hand, for rapidly emerging but risky new industries, M&A can help companies quickly acquire the resources and capabilities they need to overcome barriers to entry and innovation [29]. In recent years, the number of M&A deals in new economy industries such as technology, media, and healthcare in China has been among the highest, and it has been the consensus of new economy companies to use M&A to seize the first opportunity and use M&A as an effective means to achieve strategic layout quickly. On the other hand, for traditional industries with overcapacity, M&A can facilitate the elimination of low-quality assets and promote the aggregation of high-quality production factors into high-quality enterprises, to further enhance innovation capacity and realize industrial transformation and upgrading. For example, the more specific coal industry, under the guidance of relevant policies, has eliminated backward production capacity through large-scale M&A restructuring, changed the scattered industrial pattern, and further improved industry concentration; M&A has also encouraged the coal industry to carry out product and technology innovation, improved the cleanliness and efficiency of coal supply, and further improved overall competitiveness. Therefore, when factors such as the supply and demand environment and technological change affect industry development, M&A is often an effective and low-cost way to respond to changes in industry structure and to carry out resource replacement [30]. At this point, a goodwill impairment policy that can support mergers and acquisitions and facilitate synergies will likely positively impact industrial development.

In summary, we propose Hypothesis 2:

H2: The change in the subsequent measurement of goodwill from amortization to impairment will promote the industry’s competitiveness.

## Study design

### Data and sample

The new corporate accounting standards implemented in China in 2007 changed the subsequent measurement of goodwill from amortization to impairment testing. Based on this, and in order to exclude the interference of various favorable M&A policies and macroeconomic restructuring implemented by the Securities Regulatory Commission in 2013, this paper selects A-share listed companies from 2001–2013 as the initial research sample, excludes financial companies, and retains the sample with no missing data for all variables, finally obtaining 18,570 company annual observations. In order to eliminate the adverse effects of extreme values on the test validity, we winsorize all continuous variables in the regression model at the 1% and 99% quartiles. M&A and firm financial data are obtained from the CSMAR database. Macro-level data such as industry and economy are obtained from the CEIC macroeconomic database, the UN Comtrade Database, and the China Industry Business Performance Database.

### Dependent variables

#### Motivation for mergers and acquisitions

Our key dependent variables are the measures that capture the motivation of firms to engage in M&A. First; we construct the measures based on the frequency of mergers and acquisitions of the company in a year (MA_freq). MA_freq equals the natural logarithm of a firm’s total number of mergers and acquisitions in a year. We construct the secondary measures MA and MA_gap. MA is a dummy variable equal to 1 if a firm has a merger or acquisition in a year and 0 otherwise. MA_gap equals the natural logarithm of the average number of days between two consecutive mergers and acquisitions in a firm in one year. A higher frequency of M&A, a higher probability of M&A, and a shorter M&A time interval indicate that a firm is more active in M&A. In constructing the M&A variable, we restrict M&A to successful M&A actions with transactions more than 1 million yuan and exclude broad forms of M&A activities such as divestitures, assets replacement, or debt restructurings, following Wu et al. (2008) [31].

#### Industrial competitiveness

In contrast to the traditional financial indicators used to measure M&A performance, we selected industry concentration (HHI), total factor productivity (TFP), and the revealed comparative advantage index (RCA) to measure industrial competitiveness as an M&A effect, which can reflect the industry’s industrial structure, productivity and international competitiveness in a relatively comprehensive way.

*Industry concentration (HHI)*. Industry concentration reflects the degree of competition among firms and is an essential indicator of industrial structure. Based on the theory of industrial organization, market M&A activities will promote the evolution and optimization of industries and form a new market structure [32]. Conversely, leading enterprises in each industry will consolidate their market position and form economies of scale through large-scale M&A; conversely, M&A can help small enterprises exit the industry and relieve overcapacity. Therefore, M&A promotes the optimization of the industry structure and will further increase the concentration of the industry. We construct industry concentration by using company sales data. The higher the value of the HHI, the stronger the industry’s competitiveness.

*Total Factor Productivity (TFP)*. According to Solow’s residual value theory [33], total factor productivity is the surplus of total output after deducting input factors and is usually interpreted as the result of technological progress. As a measure of a firm’s productivity, TFP is a comprehensive reflection of an industry’s strengths in economies of scale and resource allocation efficiency. Therefore, TFP can be an appropriate measure of industrial competitiveness. The goodwill impairment policy can encourage companies to acquire scarce and high-quality assets actively, raise the industry’s innovation capacity and technology level and thus improve total factor productivity. Referring to the research methods of Giannetti et al. (2015) [34] and Lu et al. (2012) [35], We estimate the total factor productivity of enterprises by the OP method using the data of listed companies and the total factor productivity of industries is summed up in the industry dimension with total assets as the weight. The higher the TFP, the more efficient production and the more competitive the industry.

*Revealed Comparative Advantage (RCA)*. The RCA index measures the share of a particular industry in the world market. A more extensive RCA index for an industry indicates that the industry’s exports have a comparative advantage relative to the average of world exports and is an essential indicator for measuring and analyzing the international competitiveness of an industry [36]. The convergence with international accounting standards after the adoption of the goodwill impairment policy in China may have changed the disadvantaged position of Chinese enterprises in international M&A due to goodwill accounting, which can facilitate cross-border M&A and help Chinese enterprises to gain international market share and enhance the international competitiveness of the industry. Commodity export trade data are taken from the UN Comtrade database, covering 192 countries and regions and 67 product categories. The higher the RCA value, the greater the comparative advantage of the industry, indicating that the industry is more competitive in the international market.

#### Model design

Hypothesis 1 predicts that firms will engage in more M&A after adopting a goodwill impairment policy. To test this prediction, we regress a firm’s M&A activities (M&A) on our key explanatory variables, along with a set of firm characteristics that previous research has found to be associated with differences in M&A activity across firms. The model is specified as follows:

$${M\&A}_{i,t}=\beta_{0}+\beta_{1}{{GW}_{\_}IT}_{t}+\gamma {Controls}_{i,t-1}+\sum Firm+\sum Industry+\varepsilon_{i,t}$$ (1)

Our key explanatory variable (${{GW}_{\_}IT}_{t}$) is a dummy variable, which equals one if the sample is in 2007 and later, 0 otherwise. First, we select the company size (Size), debt ratio (Leverage), return on assets (ROA), cash flow (CFO), Tobin’s q ratio (TobinQ), and equity concentration (LrgHold) to control for financial characteristics of the company. Second, we construct Dual and ExcuHold to control corporate governance factors. Finally, we construct the SOE based on corporate ownership property [37]. To circumvent the effect of M&A on current firm characteristics, we lag the time-varying control variables by one period. We also control for firm and industry fixed effects in the model, and the definitions of the relevant variables are specified in Table 2.

**Table 2:** Variable definitions.

Variable	Definitions
**GW_IT**	A dummy variable, which equals one if the sample in 2007 and later, and 0 otherwise.
**MA_freq**	The natural logarithm of the total number of mergers and acquisitions that occurred in a firm in a year
**MA**	A dummy variable, which equals 1 if a firm has a merger or acquisition in a year and 0 otherwise
**MA_gap**	The natural logarithm of the average number of days between two consecutive M&A of a company in one year. The variable is missing if the enterprise never had a merger or had only one merger during the sample period.
**HHI**	Herfindahl-Hirschman index based on the top five companies in terms of sales in the industry (Unless otherwise stated, the industries in this article are classified according to the Shenyin Wanguo Industry Classification Standard (2014 edition))
**TFP**	The total factor productivity of each firm is calculated by the OP method using data from listed companies and summed up using total assets as weights to obtain the total factor productivity of the industry.
**RCA**	The calculation formula is ${RCA}_{j}=(X_{j}^{c}/X^{c})÷(X_{j}^{w}/X^{w})$, where X is total annual exports, X_j_ is total annual exports for industry j (According to the standards of the National Industries Classification (GB/T4754-2017)), c and w denote China and the world.
**Size**	The natural logarithm of the company’s total assets at the end of the year.
**Leverage**	The ratio of total liabilities to total assets of the company at the end of the year.
**ROA**	The company’s annual profit after tax divided by total assets at the end of the year
**CFO**	The company’s annual cash flow from operating activities is divided by total assets at the end of the year.
**TobinQ**	The sum of the market value of the company’s equity and debt divided by total assets at the end of the year.
**ExcuHold**	The total number of shares held by senior executives divided by total share capital.
**Dual**	A dummy variable that is equal to 1 if there is a duality of the chairman of the board and the CEO in the company; otherwise, it is 0.
**LrgHold**	Total number of shares held by the largest shareholder divided by total share capital.
**SOE**	A dummy variable is equal to 1 if the nature of the actual controller in the company is the state and 0 otherwise.
**Size_ind**	Average log assets of companies in the industry
**ROA_ind**	The average return on assets of companies in the industry
**Lev_ind**	The average debt ratio of companies in the industry
**Techin**	Total R&D expenditure as a percentage of sales for companies in the industry
**Subsidy**	The natural logarithm of the average amount of government subsidies to companies in the industry
**GDPr**	Gross Domestic Product (GDP) growth rate, (current period GDP—previous period GDP)/previous period GDP

Our Hypothesis 2 predicts that industry competitiveness will improve after adopting a goodwill impairment policy. We test this prediction using the following model:

$${Competitiveness}_{j,t}=\beta_{0}+\beta_{1}{{GW}_{\_}IT}_{t}+\gamma {Controls\_ind}_{j,t-1}+\sum Industry+\varepsilon_{j,t}$$ (2)

We control for other industry-level factors that may affect industry competitiveness (Controls_ind), such as industry size (Size_ind), industry return on assets (ROA_ind), industry leverage (Lev_ind), industry technology level (Techin), government subsidies (Subsidy), and GDP growth rate (GDPr) to control for country-level factors, based on existing studies [38]. All industry regression models control for industry-fixed effects. The relevant variables are specified in Table 2.

#### Data description

Table 3 reports the results of descriptive statistics for the main variables; Panels A and B are based on two different samples, one for the M&A analysis and the other for the industrial Competitiveness analysis. GW_IT in Panel A is 0.625, indicating that 62.5% of the sample is located in the goodwill impairment testing period. Given the right-skewed distribution of the MA_freq and MA_gap, we apply the natural logarithm to these two indicators, and MA_freq1 and MA_gap1 show the original actual values. As shown in Panel A, more than half of the sample has experienced mergers and acquisitions, and the average annual frequency of mergers and acquisitions for companies is 1.56, with an average interval of 305 days between two consecutive M&As, indicating that M&As are common among Chinese listed companies. The standard deviation indicates that the motivation for M&A varies considerably between companies. As shown in Panel B, the mean value of the RCA index is 1.045, indicating that Chinese goods have a comparative advantage in the international market and that the relevant industries have some international competitiveness.

**Table 3:** Summary statistics for main variables.

**A. Summary statistics for model (1)**						
**Variables**	**N**	**Mean**	**SD**	**Min**	**Median**	**Max**
**GW_IT**	18,570	0.625	0.484	0.000	1.000	1.000
**MA_freq1**	18,570	1.560	2.536	0.000	1.000	14.000
**MA_freq**	18,570	0.634	0.718	0.000	0.693	2.708
**MA**	18,570	0.531	0.499	0.000	1.000	1.000
**MA_gap1**	9,291	304.742	349.438	0.000	182.5	1,864
**MA_gap**	9,291	5.073	1.352	0.000	5.212	7.531
**Size**	18,570	21.418	1.146	18.751	21.267	25.101
**Leverage**	18,570	0.479	0.251	0.048	0.473	1.890
**ExcuHold**	18,570	0.029	0.096	0.000	0.000	0.549
**Dual**	18,570	0.411	0.492	0.000	0.000	1.000
**LrgHold**	18,570	0.387	0.162	0.092	0.367	0.758
**ROA**	18,570	0.030	0.073	-0.378	0.036	0.211
**CFO**	18,570	0.045	0.081	-0.219	0.045	0.277
**TobinQ**	18,570	1.741	0.982	0.940	1.416	7.136
**SOE**	18,570	0.646	0.478	0.000	1.000	1.000
**B. Summary statistics for model (2)**						
**Variables**	**N**	**Mean**	**SD**	**Min**	**Median**	**Max**
**GW_IT**	1219	0.561	0.496	0.000	1.000	1.000
**HHI**	1009	0.279	0.109	0.201	0.242	0.851
**TFP**	1119	5.253	0.514	2.175	5.257	6.108
**RCA**	452	1.045	0.918	0.018	0.745	3.667
**Size_ind**	1219	21.460	0.845	19.441	21.282	24.792
**Lev_ind**	1219	0.537	0.260	0.173	0.485	1.836
**ROA_ind**	1219	0.021	0.074	-0.364	0.033	0.168
**Techin**	1219	0.011	0.025	0.000	0.003	0.144
**Subsidy**	1219	1.462	2.479	0.000	0.688	22.719
**GDPr**	1219	0.101	0.018	0.080	0.100	0.140

## Empirical results

### Regression results of M&A motivation

Table 4 reports the baseline regression results of the goodwill impairment policy affecting the firm’s M&A activities. As predicted, the goodwill impairment policy (GW_IT) coefficients are all significant at the 1% level in Columns 1–3. These results indicate that the goodwill impairment test increases the probability of firm M&A, increases the frequency of firm M&A, and shortens the time interval between consecutive M&A relative to amortization, and are consistent with the prediction of our career concerns hypothesis of the positive relationship between goodwill impairment policy and firm’s M&A motivation. The coefficients on control variables are primarily consistent with previous studies [39]. For example, the larger and more growth-oriented (higher TobinQ) private companies are more motivated to acquire.

**Table 4:** Regression results of M&A motivation.

	(1)	(2)	(3)
	MA_freq	MA	MA_gap
**GW_IT**	0.305*** (16.20)	0.985***	-0.228***
		(14.54)	(-4.33)
**Size**	0.059***	0.086*	-0.019 (-0.59)
	(4.48)	(1.89)	
**Leverage**	-0.038	-0.256	0.376***
	(-0.87)	(-1.64)	(3.37)
**ExcuHold**	-0.460**	-1.617*	-2.360***
	(-2.18)	(-1.93)	(-3.37)
**Dual**	-0.009	0.030	0.001
	(-0.56)	(0.50)	(0.01)
**LrgHold**	-0.043 (-0.51)	-0.350	-0.326
		(-1.18)	(-1.57)
**ROA**	-0.047	0.012	-0.585**
	(-0.47)	(0.03)	(-2.44)
**CFO**	0.002	0.107	0.613***
	(0.03)	(0.39)	(3.20)
**TobinQ**	0.018**	0.025	-0.070***
	(2.22)	(0.93)	(-3.79)
**SOE**	-0.077***	-0.100	-0.147**
	(-2.91)	(-1.06)	(-1.98)
**Constant**	-0.749***	-2.592**	5.884***
	(-2.67)	(-2.25)	(8.50)
**Firm**	Yes	Yes	Yes
**Industry**	Yes	Yes	Yes
**N**	18570	17084	9291
**Adj_R2**	0.22	0.15	0.17

Columns 1 and 3 present OLS regression results and Column 2 reports the logistic regression results for the firm’s M&A probability. T-statistics in parentheses are based on heteroscedasticity-consistent standard errors clustered at the firm level. ***, **, and * indicate significance at the 1%, 5% and 10% levels respectively.

### Regression results of industrial competitiveness

We examine the impact of goodwill impairment on industry competitiveness from three aspects, industry concentration (HHI), productivity (TFP), and international competitiveness (RCA), according to the industry model (2), and the regression results are reported in Table 5. The coefficients on the explanatory variables in Columns 1 and 2 are both significantly positive at the 1% level. The dependent variable in column 3 is the RCA index, where the coefficient on GW_IT is significantly positive at the 5% level. These results indicate that the goodwill impairment policy can promote industrial restructuring, increase industry concentration, and improve industry productivity and competitive advantage in the international market. These results are also consistent with the prediction of our hypothesis of the positive relationship between goodwill impairment policy and industry competitiveness.

**Table 5:** Regression results of industry competitiveness.

	(1)	(2)	(3)
	HHI	TFP	RCA
**GW_IT**	0.026***	0.230***	0.081**
	(4.88)	(8.92)	(2.40)
**Size_ind**	-0.009*	0.240***	0.079**
	(-1.66)	(8.91)	(2.34)
**Lev_ind**	0.003	-0.010	-0.035
	(0.29)	(-0.19)	(-0.33)
**ROA_ind**	0.000	-0.027	-0.028
	(0.01)	(-0.16)	(-0.13)
**Techin**	-0.386***	-1.605***	-0.845
	(-4.29)	(-3.11)	(-1.08)
**Subsidy**	-0.004	0.007	-0.010
	(-1.31)	(1.15)	(-1.15)
**GDPr**	0.107	-1.092*	-0.107
	(1.05)	(-1.94)	(-0.16)
**Constant**	0.454*** (3.84)	0.127 (0.22)	-0.640 (-0.89)
**Industry**	Yes	Yes	Yes
**N**	1009	1119	452
**Adj_R2**	0.77	0.63	0.93

The dependent variables in columns 1–3 are the industry concentration (HHI), total factor productivity (TFP), and revealed comparative advantage (RCA), respectively. The explanatory variable is GW_IT. Columns 1–3 present OLS regression results. ***, **, and * indicate significance at the 1%, 5% and 10% levels respectively.

## Additional analysis

### Heterogeneity among firms

In our hypothesis analysis, we propose that the mechanism by which the implementation of a goodwill impairment policy can motivate firms to engage in M&A is that the goodwill impairment policy avoids the forced amortization of goodwill, which hurts post-merger performance, and leads firms to have better expectations of M&A performance. Under this mechanism, some companies that are expected to generate more goodwill in their M&A activities will experience a more significant negative impact on their post-merger profits due to the mandatory amortization policy. As a result, implementing a goodwill impairment policy will exert a more substantial influence on the M&A activities of these particular companies. To verify this effect mechanism, we perform a subsample test, expecting to observe a more significant effect of the goodwill impairment policy among companies expected to generate more M&A goodwill.

We suggest that companies with light assets or high P/E ratios are expected to generate more goodwill in their M&A activities, and changes in goodwill accounting will inevitably have a more significant impact on these companies. First, in terms of M&A premiums, M&A in asset-light industries generates higher premiums. Asset-light companies invest less in tangible assets such as fixed assets and have more miniature balance sheets, so the book value does not accurately reflect the overall value of the company, and such unidentifiable assets are usually of high scarcity and value, resulting in high goodwill on acquisitions of such companies. Such unidentifiable assets are usually scarce and valuable, resulting in high goodwill on acquisitions of asset-light companies. In addition, in terms of M&A motivation, the price-earnings ratio reflects a firm’s long-term growth, and firms with higher price-earnings ratios tend to have more substantial capital expansion capabilities and M&A motivation [40], so these firms will engage in large-scale M&A more frequently and generate more goodwill. Therefore, we expect to observe a stronger impact of the accounting treatment of goodwill on firms with asset-light or high P/E class characteristics.

Specifically, we define “light-asset” firms as those whose ratio of fixed assets to total assets in a given year is located in the bottom 30% of the sample, and the top 30% are heavy-asset firms. The companies with a price/earnings ratio in a given year in the top 30% of the sample are classified as high P/E groups, while the bottom 30% are classified as low P/E groups. Table 6 reports the results of the analysis of the effect of goodwill impairment policy across firms. In Panel A, we report the results for the partition based on the light-assets characteristic. In columns 1–4, the coefficients of *GW_IT* are all statistically significant at the 1% level, with the coefficients of *GW_IT* in the subsample with light-asset firms being all greater than those in the subsample with heavy-asset firms and the different tests of the coefficients of *GW_IT* between the subsamples are statistically significant. The results in columns 5 and 6 show that the effect of goodwill impairment on *MA_gap* is significantly negative at the 5% level for both subsamples and there is no significant difference between the coefficients of *GW_IT* between the two groups. Overall, the results indicate that the impact of goodwill impairment testing is more pronounced in the subsample of light-asset firms.

**Table 6:** The effect of goodwill impairment policy across firms.

**A. Analysis partitioned by Light-Asset characteristic.**						
	**(1)**	**(2)**	**(3)**	**(4)**	**(5)**	**(6)**
	**MA_freq**	**MA_freq**	**MA**	**MA**	**MA_gap**	**MA_gap**
**Partition Var.**	**Light-Asset**	**Heavy-Asset**	**Light-Asset**	**Heavy-Asset**	**Light-Asset**	**Heavy-Asset**
**GW_IT**	0.391***	0.288***	1.214***	0.930***	-0.238**	-0.239**
	(10.10)	(8.00)	(8.06)	(6.70)	(-2.27)	(-2.53)
**Size**	0.127***	-0.008	0.297***	-0.049	-0.092	-0.073
	(4.35)	(-0.30)	(2.97)	(-0.48)	(-1.47)	(-1.14)
**Leverage**	-0.162	-0.040	-0.749**	-0.342	0.613**	0.400
	(-1.57)	(-0.44)	(-2.18)	(-0.97)	(2.37)	(1.43)
**ExcuHold**	0.362	-0.907*	0.632	-5.250	-2.008	-1.483
	(1.00)	(-1.72)	(0.39)	(-1.61)	(-1.01)	(-0.76)
**Dual**	-0.003	-0.049*	0.026	-0.065	0.070	0.000
	(-0.09)	(-1.80)	(0.18)	(-0.59)	(0.77)	(0.01)
**LrgHold**	-0.039	-0.031	0.619	-1.012	0.341	-0.987**
	(-0.21)	(-0.19)	(0.94)	(-1.53)	(0.94)	(-2.26)
**ROA**	0.051	-0.191	0.397	-0.642	-0.997*	-0.677
	(0.25)	(-0.99)	(0.50)	(-0.82)	(-1.93)	(-1.22)
**CFO**	0.056	-0.028	0.166	0.123	0.320	0.656
	(0.45)	(-0.17)	(0.34)	(0.17)	(1.20)	(1.39)
**TobinQ**	0.049**	0.003	0.172***	-0.022	-0.090***	-0.155***
	(2.52)	(0.17)	(2.58)	(-0.36)	(-2.75)	(-3.54)
**SOE**	-0.137**	-0.022	-0.188	-0.069	-0.082	-0.228
	(-2.47)	(-0.36)	(-0.83)	(-0.31)	(-0.54)	(-1.62)
**Constant**	-2.165***	0.622	-8.044***	0.578	6.932***	7.609***
	(-3.50)	(1.13)	(-3.27)	(0.20)	(5.34)	(5.31)
**Firm**	Yes	Yes	Yes	Yes	Yes	Yes
**Industry**	Yes	Yes	Yes	Yes	Yes	Yes
**N**	5011	5345	4138	4844	2508	2315
**Adj_R2**	0.28	0.19	0.18	0.16	0.18	0.13
**Difference in GW_IT**	Diff = 0.103**		Diff = 0.284**		Diff = 0.001	
	Chi-square = 3.87		Chi-square = 5.58		Chi-square = 0.08	
**B. analysis partitioned by P/E ratio**						
	**(1)**	**(2)**	**(3)**	**(4)**	**(5)**	**(6)**
	**MA_freq**	**MA_freq**	**MA**	**MA**	**MA_gap**	**MA_gap**
**Partition Var. P/E ratio**	**High**	**Low**	**High**	**Low**	**High**	**Low**
**GW_IT**	0.369***	0.224***	1.254***	0.788***	-0.398***	-0.059
	(10.54)	(6.18)	(7.74)	(5.31)	(-3.51)	(-0.62)
**Size**	0.023	0.078***	0.071	0.146	0.040	-0.186***
	(0.80)	(3.10)	(0.55)	(1.58)	(0.45)	(-3.04)
**Leverage**	-0.151*	-0.053	-0.659*	-0.290	0.745***	0.185
	(-1.86)	(-0.72)	(-1.84)	(-1.01)	(2.94)	(0.92)
**ExcuHold**	0.222	-0.536	-0.020	-4.582	-1.199	1.902
	(0.50)	(-0.59)	(-0.01)	(-1.16)	(-1.07)	(1.56)
**Dual**	-0.006	-0.030	0.068	-0.090	-0.123	0.006
	(-0.19)	(-0.95)	(0.50)	(-0.69)	(-1.18)	(0.07)
**LrgHold**	0.259	-0.265*	0.735	-0.970	0.343	0.601*
	(1.54)	(-1.66)	(0.97)	(-1.60)	(0.69)	(1.68)
**ROA**	-0.998	-0.039	0.542	-0.479	-0.346	-0.079
	(-1.41)	(-0.26)	(0.17)	(-0.79)	(-0.18)	(-0.20)
**CFO**	0.114	-0.091	0.933	0.007	0.889*	0.326
	(0.72)	(-0.64)	(1.37)	(0.01)	(1.89)	(0.95)
**TobinQ**	0.012	0.035*	0.053	0.119*	-0.032	-0.120***
	(0.82)	(1.90)	(0.91)	(1.74)	(-0.97)	(-2.97)
**SOE**	-0.067	-0.115**	-0.064	-0.161	-0.242	0.194
	(-1.34)	(-2.33)	(-0.32)	(-0.81)	(-1.45)	(1.52)
**Constant**	-0.096	-1.022*	-3.812	-3.506	4.345**	9.079***
	(-0.16)	(-1.84)	(-1.25)	(-1.34)	(2.35)	(6.62)
**Firm**	Yes	Yes	Yes	Yes	Yes	Yes
**Industry**	Yes	Yes	Yes	Yes	Yes	Yes
**N**	5,122	5,165	4,256	4,135	2,083	2,335
**Adj_R2**	0.22	0.22	0.15	0.16	0.17	0.17
**Difference in GW_IT**	Diff. = 0.145**		Diff. = 0.466***		Diff. = -0.339**	
	Chi-square = 4.44		Chi-square = 8.96		Chi-square = 5.77	

Panel A reports the results for the partition based on the light-assets characteristic; Panel B reports the results for the partition based on the P/E ratio. The models use MA_freq, MA, and MA_gap as the dependent variables. Columns 1–2 and 5–6 present OLS regression results, and Columns 3–4 report the logistic regression results for the firm’s M&A probability. Coefficient difference tests are based on the seemingly unrelated regressions SUR.t-statistics in parentheses are based on heteroscedasticity-consistent standard errors clustered at the firm level. ***, **, and * indicate significance at the 1%, 5% and 10% levels respectively.

In Panel B, we report the results of the partition based on the P/E ratio of firms. The results in columns 1–4 show that goodwill impairment (GW_IT) has a significant positive effect on M&A frequency and M&A probability in all subsamples, with the coefficient of GW_IT being greater in the high P/E group than in the low P/E group, the difference tests of the coefficients of GW_IT between the subsamples are statistically significant. Columns 5 and 6 report the subsample regression results with MA_gap as a dependent variable. For the high P/E subsample, the coefficient of GW_IT is statistically significant at the 1 percent level. For the low P/E subsample, the corresponding coefficient is statistically indistinguishable from zero. The overall results suggest that the goodwill impairment policy has a more significant effect on the M&A motivation when the firm has high P/E characteristics and is consistent with the view that the goodwill impairment policy influences M&A activities by reduction of profit loss.

### Heterogeneity among industries

Similarly, industries with asset-light or high P/E ratio characteristics tend to have high growth potential and generate a large amount of goodwill from M&A during the industrial development process, so goodwill impairment may have a more significant impact on the M&A development activities of these two types of industries. Therefore, compared with other industries, goodwill impairment policies significantly impact the competitiveness of asset-light industries or industries with high P/E ratios. Specifically, we separately rank the industry’s fixed asset ratio and P/E ratio in a given year. We define "light-asset" industries as those whose ratio of fixed assets to total assets in a given year is located in the bottom 30% of the sample, and the top 30% are heavy-asset industries. The industries with a price/earnings ratio in a given year in the top 30% of the sample are classified as high P/E groups, while the bottom 30% are classified as low P/E groups.

Table 7 shows the analysis of the effect of the goodwill impairment policy on industry competitiveness across industries. In Panel A, we report the results for the partition based on the industries’ light-assets characteristics. Columns 1–2 and 5–6 report the results for subsamples where the dependent variable is industry concentration (HHI) and RCA index. For the subsamples of industries with light-assets characteristics (Column1, Column5), the coefficients of *GW_IT* are statistically significant at the 1 percent level. By contrast, for the subsample of heavy-asset industries, the coefficient of *GW_IT* is statistically insignificant. The results in columns 3 and 4 show that goodwill impairment policy has a significant positive effect on TFP for both subsamples, with the coefficient of *GW_IT* being greater in the light-asset group than in the high-asset group, the different tests of the coefficients of *GW_IT* between the subsamples are statistically significant. The above results indicate that goodwill impairment policies can accommodate the high premium M&A needs of the light-asset industries compared to the heavy-asset industries. They are more likely to facilitate their industrial restructuring, optimization of production efficiency, and acquisition of international market share.

**Table 7:** The Effect of goodwill impairment policy across industries.

**A. Analysis partitioned by industrial light-asset characteristic.**						
	(1)	(2)	(3)	(4)	(5)	(6)
	HHI	HHI	TFP	TFP	RCA	RCA
Partition Var.	Light-Asset	Heavy-Asset	Light-Asset	Heavy-Asset	Light-Asset	Heavy-Asset
GW_IT	0.025**	0.012	0.396***	0.207***	0.373***	-0.019
	(2.19)	(1.31)	(5.07)	(7.13)	(4.21)	(-0.31)
Size_ind	0.013	-0.022***	0.165**	0.175***	0.144*	0.064
	(1.12)	(-2.75)	(2.10)	(6.22)	(1.92)	(1.21)
Lev_ind	0.024	-0.019	-0.081	-0.013	0.009	-0.177
	(1.20)	(-0.93)	(-0.48)	(-0.21)	(0.04)	(-1.00)
ROA_ind	0.001	-0.011	-0.073	0.025	-0.184	0.035
	(0.01)	(-0.23)	(-0.15)	(0.14)	(-0.36)	(0.09)
Techin	-0.579***	-0.259	-1.630	-0.057	0.635	-2.634
	(-4.16)	(-0.48)	(-1.47)	(-0.03)	(0.39)	(-0.39)
Subsidy	0.003	-0.004	0.034*	-0.016**	-0.051**	-0.002
	(0.41)	(-0.78)	(1.82)	(-2.43)	(-2.16)	(-0.15)
GDPr	-0.010	0.426***	-4.491***	1.137**	1.769	0.176
	(-0.05)	(2.71)	(-2.68)	(2.08)	(0.95)	(0.18)
Constant	-0.018	0.718***	2.010	1.358**	-1.964	-0.558
	(-0.07)	(4.11)	(1.18)	(2.25)	(-1.23)	(-0.49)
Industry	Yes	Yes	Yes	Yes	Yes	Yes
N	295	288	330	330	130	133
Adj_R2	0.73	0.76	0.45	0.81	0.89	0.90
Difference in GW_IT	Diff. = 0.013		Diff. = 0.189**		Diff. = 0.392***	
	Chi-square = 0.96		Chi-square = 5.94		Chi-square = 23.94	

**B. Analysis partitioned by industrial P/E ratio.**				
	(1)	(2)	(3)	(4)
	HHI	HHI	TFP	TFP
Partition Var. P/E ratio	High	Low	High	Low
GW_IT	0.025**	0.027	0.161***	0.122
	(2.50)	(1.56)	(3.98)	(1.58)
Size_ind	-0.016	0.006	0.267***	0.396***
	(-1.31)	(0.46)	(5.66)	(5.66)
Lev_ind	0.013	-0.061	0.053	-0.218
	(0.82)	(-1.44)	(0.76)	(-0.90)
ROA_ind	0.030	-0.028	0.102	-0.191
	(0.71)	(-0.21)	(0.55)	(-0.26)
Techin	-0.283*	-0.838**	-2.835***	-2.155
	(-1.95)	(-2.18)	(-4.72)	(-0.90)
Subsidy	-0.012**	-0.004	0.050***	-0.020
	(-2.03)	(-0.52)	(3.86)	(-1.41)
GDPr	0.147	0.138	-1.315	-3.216**
	(0.84)	(0.45)	(-1.61)	(-2.03)
Constant	0.586**	0.174	-0.459	-2.803*
	(2.18)	(0.59)	(-0.46)	(-1.86)
Industry	Yes	Yes	Yes	Yes
N	251	266	273	331
Adj_R2	0.67	0.67	0.84	0.43
Difference in GW_IT	Diff. = -0.002			Diff. = 0.039		
	Chi-square = 0.01			Chi-square = 0.29		

Panel A reports the results for the partition based on the industrial light-asset characteristic, using HHI, TFP, and RCA as the dependent variables. Panel B reports the results of the partition based on the P/E ratio, using HHI and TFP as the dependent variables. Coefficient difference tests are based on the seemingly unrelated regressions SUR. ***, **, and * indicate significance at the 1%, 5% and 10% levels respectively.

In Panel B, we report the results of the partition based on the P/E ratio of industries. In the regression models where the dependent variable is the RCA index, we use data on unlisted companies and do not have a P/E ratio indicator. Only the regression results with the dependent variables HHI and TFP are reported in Panel B. We obtain statistically significant estimates of the GW_IT coefficient for the high P/E subsample in Columns 1 and 3. By contrast, the corresponding coefficients for the low P/E subsample in Columns 2 and 4 are statistically insignificant. The results indicate that goodwill impairment policies can accommodate the need for large-scale M&A in high-growth industries and thus help such industries achieve their restructuring and productivity optimization goals.

## Robustness checks

### Time trend effect

In model (1), we control for firm and industry fixed effects and use the coefficient of GW_IT to test the impact of impairment policy on M&A, where the findings may become less robust due to the presence of the time trend effect in M&A activity itself. To alleviate this problem and test whether there was already a time trend effect of M&A activities before the goodwill impairment policy was implemented, we set the year dummy indicators with 2001 as the base year to replace *the GW_IT* indicator. *2002_year* is a dummy variable for 2002. Then, we re-estimate model (1) after replacing the *GW_IT* indicator with the twelve dummy variables for 2002–2013. The results in Table 8 show that the coefficients of 2002_year, 2003_year, 2004_year, and 2005_year are not significantly positive in the regression models with dependent variable MA_freq, which indicates that the company’s M&A activities did not change significantly from 2001 to 2005, and there is no significant time trend issue. The results also show a small "jump" in M&A activity in 2006 because the new goodwill accounting policy implemented in 2007 was issued in 2006, and partial M&A goodwill generated by listed companies in 2006 was subsequently measured using the impairment test method.

**Table 8:** Test of time trend effect.

	(1)
	MA_freq
2002_year	-0.008
	(-0.38)
2003_year	0.012
	(0.52)
2004_year	-0.015
	(-0.64)
2005_year	-0.041*
	(-1.67)
2006_year	0.056**
	(2.05)
2007_year	0.339***
	(10.95)
2008_year	0.279***
	(8.91)
2009_year	0.368***
	(11.15)
2010_year	0.256***
	(7.47)
2011_year	0.172***
	(4.98)
2012_year	0.152***
	(4.51)
2013_year	0.275***
	(7.70)
Size	0.101***
	(6.64)
Leverage	-0.055
	(-1.21)
ExcuHold	-0.450**
	(-2.10)
Dual	-0.001
	(-0.08)
LrgHold	-0.080
	(-0.94)
ROA	-0.021
	(-0.21)
CFO	-0.082
	(-1.10)
TobinQ	0.047***
	(4.87)
SOE	-0.081***
	(-3.02)
Constant	-1.642***
	(-5.10)
Firm	Yes
Industry	Yes
N	18570
Adj_R2	0.23

The dependent variable is the MA_freq. The explanatory variable is the year dummy indicators from 2002 to 2013. Column 1 presents OLS regression results. T-statistics in parentheses are based on heteroscedasticity-consistent standard errors clustered at the firm level. ***, **, and * indicate significance at the 1%, 5% and 10% levels respectively.

### M&A frequency trend chart

**Fig 1:**
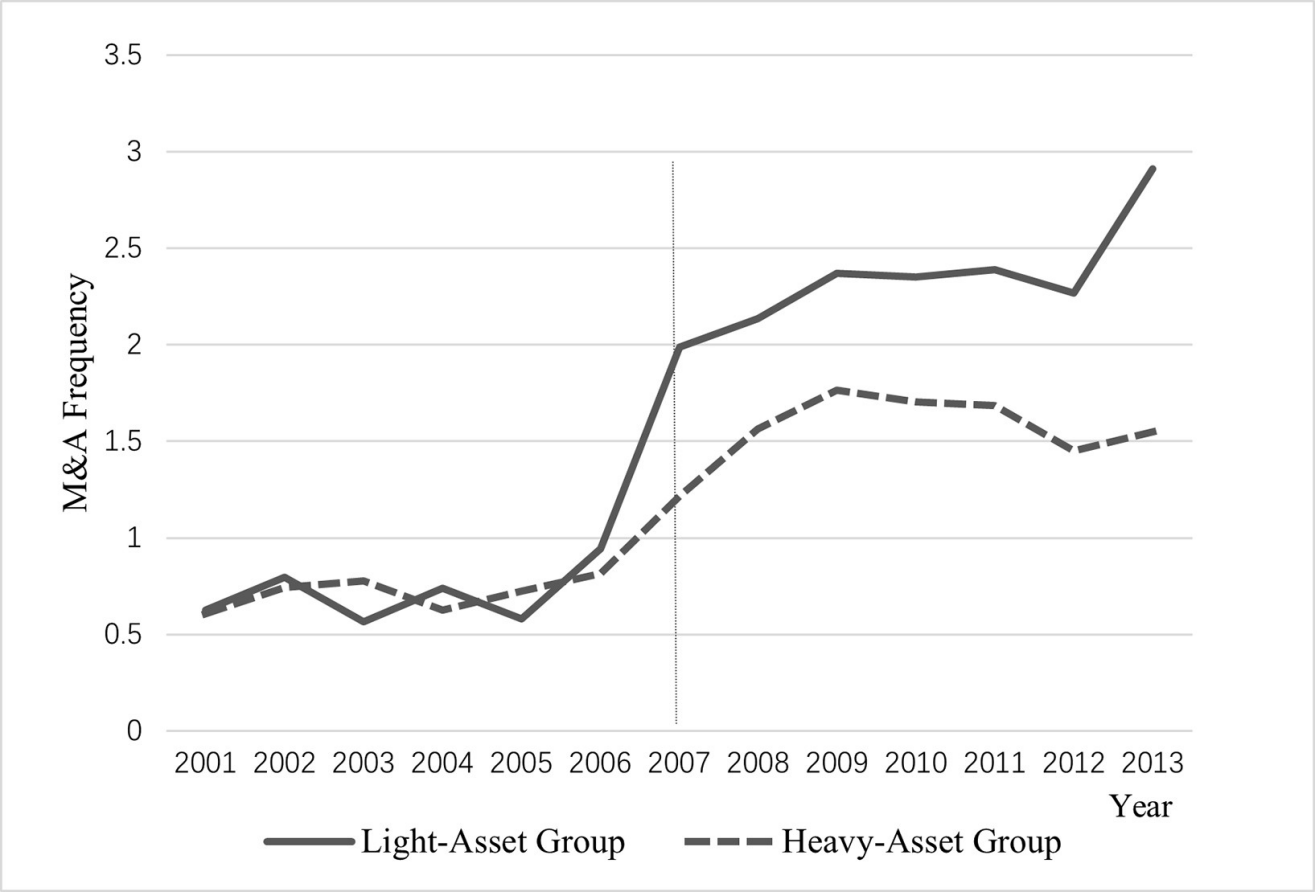
The trend of M&A frequency for subsamples partitioned by light-asset characteristic.

**Fig 2:**
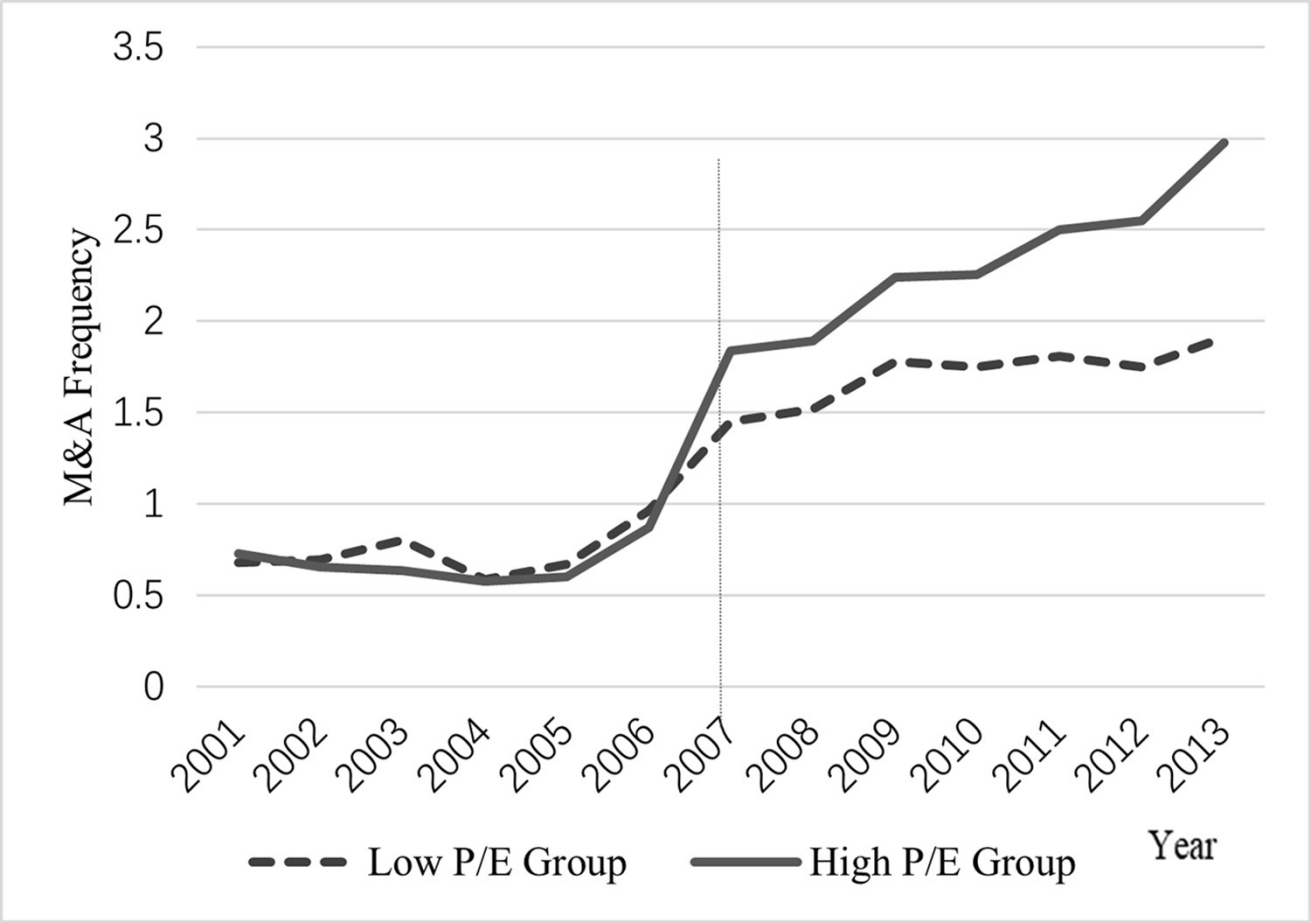
The trend of M&A frequency for subsamples partitioned by P/E ratio.

The results of the previous cross-sectional tests suggest that changes in goodwill policy have a more significant impact on firms with asset-light characteristics or high price-to-earnings ratios. Moreover, if such firms differ from other firms in terms of M&A activity in a non-parallel way, the conclusions are less robust. To alleviate this problem, we plot the trend in the average M&A frequency for the subsamples based on the partition of asset-light and price-to-earnings characteristics. As shown in Figs 1 and 2, prior to the implementation of the goodwill impairment policy in 2007, there were no significant differences in M&A frequency between the light-asset group and the heavy-asset group, and between the high P/E group and the low P/E group, thus eliminating the concern of non-parallel differences. Moreover, after the change from goodwill amortization to impairment, there is a big jump in M&A frequency for each group, with the light asset group and the high P/E group showing a more substantial increase.

### Alternative measures of industrial competitiveness

In addition, we replace the TFP and RCA indicators and re-estimate the industrial competitiveness model. First, based on Lu et al. (2012) [35], we used the fixed effects approach to replace the OP method to re-estimate the total factor productivity to construct the new variable *TFP1*. Second, we used data from China Industry Business Performance Database to construct the new variable of total factor productivity indicator *TFP2*. In addition, we use industry value-added data from the World Input-Output Database (WIOD) to construct the CA index, which replaces the export date in the RCA index. The regression results are shown in Table 9. The coefficients of *GW_IT* in Columns 1–3 are significantly positive, indicating that the results have not changed and confirming the reliability of the relevant findings.

**Table 9:** Alternative measures of industrial competitiveness.

	(1)	(3)	(3)
	TFP1	TFP2	CA
**GW_IT**	0.188***	0.605***	0.044*
	(10.18)	(10.88)	(1.72)
**Size_ind**	0.124***	0.182***	0.009
	(6.43)	(3.41)	(0.30)
**Lev_ind**	-0.033	-0.422***	0.017
	(-0.85)	(-2.71)	(0.71)
**ROA_ind**	0.019	-0.025	-0.109
	(0.16)	(-0.06)	(-0.73)
**Techin**	-1.597***	1.134	-0.386
	(-4.32)	(0.76)	(-1.02)
**Subsidy**	0.005	-0.044***	-0.003
	(1.18)	(-3.82)	(-0.38)
**GDPr**	-0.814**	2.008*	1.493***
	(-2.01)	(1.83)	(2.88)
**Constant**	0.543	4.542***	-0.438
	(1.31)	(4.00)	(-0.67)
**Industry**	Yes	Yes	Yes
**N**	1119	334	291
**Adj_R2**	0.61	0.91	0.32

The dependent variables in columns 1–3 are the TFP1, TFP2, and CA, respectively. ***, **, and * indicate significance at the 1%, 5% and 10% levels respectively.

## Conclusions

With the new economy opening up, many companies have integrated resources through mergers and acquisitions, seeking core competencies such as technology and management capital while generating vast amounts of goodwill. However, goodwill impairment can quickly become a tool for management to manipulate profits, and the enormous amount of goodwill hides a greater risk of M&A. How goodwill is subsequently measured is a critical issue. In 2007, the new accounting standards in China changed the subsequent measurement of goodwill from amortization to impairment testing. This paper takes advantage of this policy change to examine the impact of the goodwill impairment policy on corporate M&A activities and industrial competitiveness, trying to answer whether goodwill should be amortized or impaired from an economic perspective. This paper breaks through the limitations of the existing literature on the subsequent measurement policy of goodwill in terms of research perspectives. It confirms that goodwill accounting has a significant impact on the investment activities of companies as well as on industrial development.

The results of this paper show that after the change in the subsequent measurement of goodwill from amortization to impairment, firms’ M&A enthusiasm increases, as evidenced by the increase in M&A frequency and M&A probability and the shortening of consecutive M&A time intervals. The goodwill impairment policy further enhances industry concentration, production efficiency, and comparative advantage in international trade and strengthens industry competitiveness. In addition, the cross-sectional test results show that the positive effect of goodwill impairment on M&A is more significant among firms with light-asset or high P/E characteristics. Also, goodwill impairment promotes industrial competitiveness more in light-asset or high P/E industries.

Based on these results, our analysis suggests that the subsequent measurement of goodwill should continue with the impairment testing approach and not revert to the old amortization path. The goodwill impairment policy has been questioned mainly because the goodwill impairment process is not transparent, which creates room for managerial opportunism and reduces the quality of accounting information. However, earnings management issues are not an inevitable drawback of goodwill impairment policies; companies with suitable governance structures can still accurately and timely reflect the value of goodwill, while the positive impact of goodwill impairment on corporate mergers and acquisitions and industrial development is direct and comprehensive. Especially in the new economy, where economic structure and asset forms are changing rapidly, and unidentifiable intangible assets account for an increasing proportion of trading activities, accounting policies need to appropriately give users more managerial discretion in order to reflect the value of relevant assets more accurately and timely, facilitating rapid corporate development. The policy of goodwill impairment can improve the company’s enthusiasm for mergers and acquisitions and promote the efficiency of allocating high-quality assets such as technology and human capital, which can enhance economic vitality and national competitiveness.

When the goodwill impairment policy is controversial in China, what we need to do is not return to the old way of amortization but continue to improve the accounting standards for the scope of goodwill recognition and the impairment steps, as well as to standardize the disclosure rules for impairment indications and impairment transactions. It should also strengthen internal and external governance to restrain management’s opportunistic behavior from avoiding and remedying the risks and drawbacks of goodwill impairment. Accurately measuring assets and improving resource allocation efficiency are accounting standards’ general objectives. Accounting standards need to adapt to the characteristics of assets in the new environment to help enterprises move up the value chain and better serve the development of the market economy.

## Supporting information

S1 Data Data_firmmodel. (XLS)

S2 Data Data_industrymodel. (XLS)

S3 Data Data_industrymodel_RCA. (XLS)

## References

[1] Lu Y, Qu X. Earnings management motivations of goodwill impairment——the empirical evidence from Chinese a− share market. Journal of Shanxi University of Finance and Economics. 2016; 38(7): 87–99. doi: 10.13781/j.cnki.1007-9556.2016.07.008

[2] Zang AY. Evidence on the trade-off between real activities manipulation and accrual-based earnings management. The Accounting Review. 2012;87(2): 675–703. doi: 10.2308/accr-10196

[3] Healy PM, Palepu KG, Ruback RS. Does corporate performance improve after mergers. Journal of Financial Economics. 1992;31(2): 135–175. doi: 10.016/0304-405X(92)90002-F

[4] Li Z, Shroff PK, Venkataraman R, Zhang IX. Causes and consequences of goodwill impairment losses. Review of Accounting Studies. 2011;16(4): 745–778. doi: 10.1007/s11142-011-9167-2

[5] Xu JC, Zhang DX, Liu HH. Does purchased goodwill information affect the cost of debt. Journal of Central University of Finance & Economics. 2017; 3: 109–118.

[6] Zhang X, Qing C, Yang D. Internal Controls and Market Reaction to Goodwill Impairment Loss-Empirical Evidence from Chinese Listed Firms. Accounting Research. 2020; 5: 3–16.

[7] Yu L, Liu Y. An empirical analysis on M&A performance of China’s listed companies. Contemporary Economics. 2004; 7: 68–74. doi: 10.3969/j.issn.1002-2848.2004.04.012

[8] Chauvin KW, Hirschey M. Goodwill, profitability, and the market value of the firm. Journal of Accounting and Public Policy. 1994;13(2): 159–180. doi: 10.1016/0278-4254(94)90018-3

[9] Jennings R, Robinson J, Thompson RB, Duvall L. The relation between accounting goodwill numbers and equity values. Journal of Business Finance and Accounting. 1996;23(4): 513–533. doi: 10.1111/j.1468-5957.1996.tb01024.x

[10] Skinner DJ. The investment opportunity set and accounting procedure choice: preliminary evidence. Journal of Accounting and Economics. 1993;16(4): 407–445. doi: 10.1016/0165-4101(93)90034-D

[11] Wiese A. Accounting for goodwill: The transition from amortization to impairment—An impact assessment. Mediatrix Accountancy Research. 2005; (13):105–120. doi: 10.1108/10222529200500007

[12] Davis ML. Differential market reaction to pooling and purchase methods. Accounting Review. 1990;696–709.

[13] AbuGhazaleh NM, Al-Hares OM, Roberts C. Accounting discretion in goodwill impairments: UK evidence. Journal of International Financial Management & Accounting. 2011;22(3): 165–204. doi: 10.1111/j.1467-646X.2011.01049.x

[14] Chen C, Kohlbeck M, Warfield T. Timeliness of impairment recognition: Evidence from the initial adoption of SFAS 142. Advances in Accounting. 2008;24(1): 72–81. doi: 10.1016/j.adiac.2008.05.015

[15] Chalmers KG, Godfrey JM, Webster JC. Does a goodwill impairment regime better reflect the underlying economic attributes of goodwill? Accounting & Finance. 2011;51(3): 634–660. doi: 10.1111/j.1467-629x.2010.00364.x

[16] Qu X, Lu Y, Zhang R. Value relevance of goodwill impairments: empirical evidence from Chinese A-share market. Research on Economics and Management. 2017; 38(3): 122–132. doi: 10.13502/j.cnki.issn1000-7636.2017.03.013

[17] Ye J, He K, Yang Q, Ye Y. Unverifiable estimates in goodwill impairment test and audit fees. Audit Research. 2016; 1: 76–84.

[18] Yang W, Song M, Feng K. M&A goodwill, investor overreaction, and stock price bubbles and crashes. China Industrial Economics. 2018; 6: 156–173. doi: 10.19581/j.cnki.ciejournal.2018.06.010

[19] Xue S, Xu P. Can analysts see through goodwill bubbles? The impact of goodwill on analysts’ forecasts. China Journal of Accounting Studies. 2021; 9(2): 195–220.

[20] Fang J. The government intervention, the nature of ownership and enterprises’ mergers & acquisitions. Management World. 2008; 9: 118–123.

[21] Bhabra HS, Huang J. An empirical investigation of mergers and acquisitions by Chinese listed companies, 1997–2007. Journal of Multinational Financial Management. 2013; 23(3): 186–207. doi: 10.1016/j.mulfin.2013.03.002

[22] Dunne KM, Rollins TP. Accounting for goodwill: A case analysis of the US, UK and Japan. Journal of International Accounting, Auditing and Taxation. 1992;1(2), 191–207. doi: 10.1016/1061-9518(92)90016-9

[23] Mueller DC. A Theory of conglomerate mergers. The Quarterly Journal of Economics.1969;83(4): 643–659.

[24] Jensen MC, Murphy KJ. Performance pay and top-management incentives. Journal of political economy. 1990;98(2), 225–264.

[25] Roll R. The hubris hypothesis of corporate takeovers. The Journal of Business. 1986;59(2): 197–216.

[26] Zhai J, Wang Y, Li D. The effect of financing choice on M&A performance: From the perspective of functional fixation. Chi. Ind. Econ. 2011; 12: 100–110. doi: 10.1086/296325

[27] Bartov E, Cheng CSA, Wu H. Overbidding in Mergers and Acquisitions: An accounting perspective. Accounting Review. 2021;96(2): 55–79. doi: 10.2308/TAR-2018-0260

[28] Bai X, Wei J. Inter-Province Mergers, Inter-Regional Resources Flow and Industrial Upgrading: An Empirical Study Based on Chinese Data. Contemporary Finance & Economics. 2017; (01): 100–109+135–136.

[29] Deng P, Yang M. Cross-border mergers and acquisitions by emerging market firms: a comparative investigation. International Business Review. 2015;24(1): 157–172. doi: 10.1016/j.ibusrev.2014.07.005

[30] Mitchell ML, Mulherin JH. The impact of industry shocks on takeover and restructuring activity. Journal of Financial Economics. 1996;41(2): 193–229. doi: 10.1016/0304-405X(95)00860-H

[31] Wu CP, Wu SN, Zheng FB. A theoretical and empirical study on manager’s behavior and performance of serial acquisitions. Management World. 2008; 7: 126–133. doi: 10.1201/9781420009521.ch3

[32] Breinlich H. Trade liberalization and industrial restructuring through mergers and acquisitions. Journal of International Economics. 2008; 76(2): 254–266. doi: 10.1016/j.jinteco.2008.07.007

[33] Solow RM. Technical change and the aggregate production function. The Review of Economics and Statistics. 1957;9(3): 312–320.

[34] Giannetti M.; Liao G.; Yu. X. The brain gain of corporate boards: Evidence from China. The Journal of Finance. 2015;70(4): 1629–1682. doi: 10.1111/jofi.12198

[35] Lu X, Lian Y. Estimation of total factor productivity of industrial enterprises in China: 1999–2007. China Economic Quarterly. 2012; 11(2): 541–558.

[36] Balassa B. Trade liberalization and "revealed" comparative advantage. The Manchester School. 1965;33(2): 99–123.

[37] Malmendier U, Tate G. Who makes acquisitions? CEO overconfidence and the market’s reaction. Journal of Financial Economics. 2008;89(1): 20–43. doi: 10.1016/j.jfineco.2007.07.002

[38] Jiang GH. Cross-border merger and acquisitions and the development of productivity: Evidence from Chinese industry panel data. World Econ. Stud. 2017; 1: 60–69.

[39] Wan LY, Hu J. Network position, governance roles of independent directors and M&A: Evidence from China’s Listed Companies. Nankai Bus. Rev. 2014; 17: 64–73. doi: 10.3969/j.issn.1008-3448.2014.02.008

[40] Zarowin P. What determines earnings-price ratios: revisited. Journal of Accounting. Auditing & Finance. 1990; 5(3).

